# Is It Back Pain or Pott’s Disease?

**DOI:** 10.7759/cureus.22834

**Published:** 2022-03-04

**Authors:** Patrícia Carvalho, Cátia Fernandes, Serzelina Cunha, Cláudia Teixeira

**Affiliations:** 1 Family Medicine, Unidade de Saúde Familiar 3 Rios - ACES Tâmega II - Vale do Sousa Sul, Penafiel, PRT

**Keywords:** hypoesthesia, spinal, tuberculosis, paresis, low back pain

## Abstract

Tuberculosis is a public health problem in Portugal. It can have extrapulmonary manifestations, with the spine being the most frequent and significant location affected within the bone area. We present the case of a 65-year-old man with left lumbar sciatic pain, 11% body weight loss, and anorexia. Later, he developed left crural hemiparesis and hypoesthesia, failing to respond to analgesia. A computerized tomography scan of the lumbar spine showed L5-S1 spondylodiscitis. The patient was admitted for study and started empirical antibiotic therapy. Due to lack of clinical and analytical response and inconclusive bone biopsy, surgical decompression of the lumbar abscess was performed, with isolation of multi-sensitive *Mycobacterium tuberculosis*. He took anti-tuberculostatic drugs for a year and did physiotherapy, fully recovering from neurological deficits due to his illness. On account of tuberculosis's prolonged and non-specific clinical presentation, a high index of clinical suspicion is needed for a well-timed diagnosis and treatment to prevent serious complications.

## Introduction

Tuberculosis (TB) continues to be a worldwide leading cause of morbidity and mortality [1,2]. It is a serious public health problem in Portugal, where TB is a high-incidence disease, particularly in the “Vale do Sousa Sul” area, which has twice the incidence rate recorded for the country and the highest value in the Northern Region [3,4].

TB is an infection usually caused by *Mycobacterium tuberculosis* and preferentially it affects the lungs. However, it may have extrapulmonary manifestations with an incidence of 3% [2]. It has been estimated that 10% of patients with extrapulmonary TB have musculoskeletal involvement, with the spine being the most commonly and significantly affected site (50% of cases) [1-3,5]. The most recent data from Portugal date from 2014 and indicate that vertebral TB is the fourth most frequent form of extrapulmonary TB, responsible for 5.9% of cases [6]. It is commonly known as tuberculous spondylodiscitis or “Pott's disease” and often affects the thoracic and lumbar spine [2,3]. Spinal TB can be challenging to diagnose, assess, and treat. The clinical picture is usually insidious and characterized by nonspecific symptoms, which can result in delayed diagnosis and increased risk of morbidity and mortality [1,3].

With this case, the authors intend to draw attention to this diagnosis, which is uncommon in primary health care, however, can present with similar clinical features as the most common diseases. The main challenge for the diagnosis of TB is consideration of the diagnosis. The early diagnosis and treatment can substantially improve the patient’s prognosis. The authors also intend to discuss the role of the family physician in the diagnosis and follow-up of this entity.

## Case presentation

The authors present the case of a 65-year-old man, resident in “Vale do Sousa Sul” area, Portugal, a former bricklayer, with relevant personal history: arterial hypertension, diabetes mellitus type II, dyslipidemia, obesity, and regular alcohol consumption. Between May and August 2016, the patient went to the emergency department (ED) and to his family doctor multiple times for persistent complaints of low back pain, radiating to the left lower limb (LLL), which worsened with movements and was partially relieved with analgesia and physical therapy. At the beginning of the condition, he performed lumbar spine radiography that revealed signs of spondylarthrosis and discopathy between L3-L4 and L5-S1. Between June and August, he had an 11% body weight loss associated with anorexia, but without fever, night sweats, cough, hemoptysis, contact history with TB, or trauma. The condition progressed significantly, culminating, in July, with the inability to walk independently (initially with crutches and later in a wheelchair), caused by the neurological deficit developed in the LLL: grade 3/5 paresis and global hypoesthesia. Concurrently, he did not respond to the conservative treatment instituted, so he performed computed tomography (CT) of the lumbar spine which showed thickening of soft tissues at the periphery of the vertebral bodies with anterior epidural extension along L5-S1, predominantly left and with radicular compromise, raising the hypothesis of a neoformative process or spondylodiscitis. With this suspicion, at the beginning of August, the patient was sent to the ED and hospitalized for an etiological study of the presented deficits.

**Figure 1:**
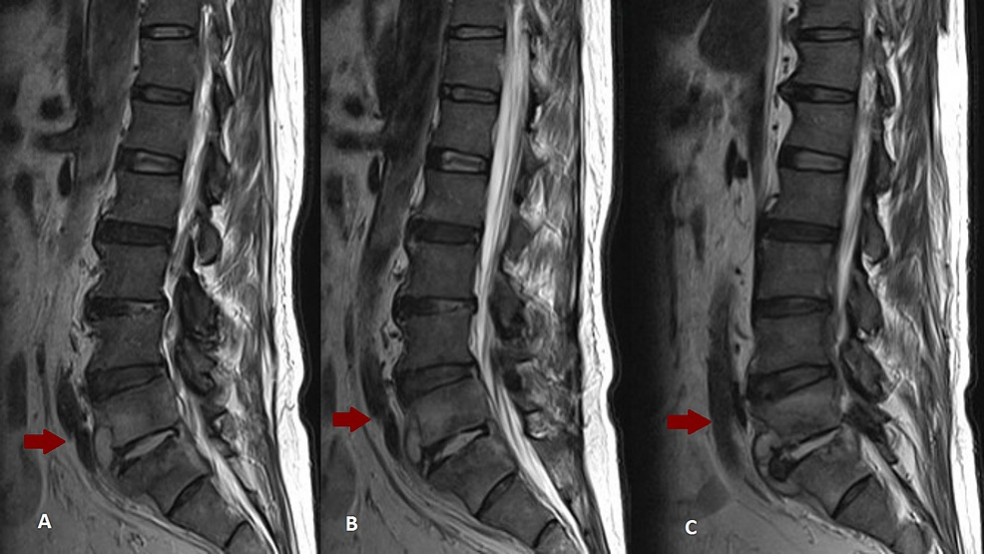
Lumbar spine MRI scan. (A-C) Multiple sagittal cuts of L5-S1 spondylodiscitis. MRI - Magnetic resonance imaging

Analytically, the blood count was normal, with a sedimentation rate of 81 mm/h and a C-reactive protein of 88 mg/L. Magnetic resonance imaging (MRI) of the lumbar spine (Figures 1A-1C) showed: signal alteration in the L5 and S1 vertebrae, evolving from hyposignal to hypersignal on T1 and T2, with frank and expressive enhancement after contrast, accompanied by alteration in the intersomatic disc, in the paravertebral and intraspinal soft tissues, constituting a mass that obliterates the anterior epidural space and the conjugation holes, with a compromise of the respective roots, strongly characteristic of spondylodiscitis. Thus, empiric antibiotic therapy with vancomycin and ceftazidime was started.

From the etiological study carried out, he presented serologies of hepatitis B and C, HIV and brucella negative, blood cultures negative, and IGRA test positive. Pulmonary TB was excluded through pulmonary CT and bronchoscopy with bronchoalveolar lavage analysis. Due to a lack of clinical and analytical response, a bone biopsy was performed, which proved to be likely contaminated. For this reason, in September, surgical decompression of the lumbar abscess was performed, isolating the sample of *M. tuberculosis *collected for culture. The sensitivity test showed that it was multi-sensitive *M. tuberculosis*. Therefore, the patient started treatment with anti-tuberculostatic drugs (isoniazid, rifampicin, pyrazinamide, and ethambutol) which he carried out for a year, having his follow-up at the Pneumological Diagnosis Center of Penafiel. Simultaneously, he performed motor rehabilitation with pain improvement and total recovery of the neurological deficits of the LLL.

## Discussion

Spondylodiscitis is an inflammation of the vertebrae, the intervertebral disk space, and adjacent soft tissue which has multifactorial etiology [7]. Among them, TB is one of its most frequent causes, particularly in the author's region [3]. Considering the significant incidence rate of TB in the “Vale do Sousa Sul” region, it is a differential diagnosis to be considered in several clinical conditions, specifically in the presence of extrapulmonary manifestations, like weight loss, anorexia, fever, and night sweats [1,4]. Spinal TB usually has an insidious onset, and the disease progresses at a slow pace, typically over four to 11 months [2,8]. The time of diagnosis, since the onset of symptoms, may differ from two weeks to several years. In the case presented, it took four months from the onset of symptoms to the definitive diagnosis.

The manifestations depend on the severity, duration, site of the disease, and the presence of complications [2]. The most common symptom is axial pain in the affected region, followed by fever and neurological deficits, like numbness and weakness with limitation of mobility [1,8]. Constitutional symptoms also include bodyweight loss, anorexia, and night sweats [1]. In complicated spinal TB, patients can present deformity, instability, and neurological deficits [2]. The patient presented a case of complicated TB with low back pain, anorexia, bodyweight loss, paresis and hypoesthesia of the LLL, and inability to walk. Yet, he never developed fever or symptoms and signs compatible with pulmonary disease, which made the diagnosis challenging.

Routine blood tests may be used to monitor and possibly exclude but not confirm the diagnosis [8]. Plain radiographs are often used as a first-line symptom study in primary health care, after a careful history and physical examination, as they are readily available. However, often demonstrate normal findings since bone destruction may not be apparent for weeks or more after the onset of symptoms. CT is better for assessing the extent of the lesion, but MRI can detect early changes and has a higher sensitivity and specificity for the diagnosis [1]. In the inability to perform an MRI, CT continues to be a relied-on exam. MRI is not available in Portuguese primary health care, thus it was performed a CT scan instead of an MRI. 

Symptoms and imaging may suggest the diagnosis, though a definitive diagnosis can only be made based on a culture of a specimen obtained by means of biopsy or aspiration [1,2]. Also, it allows establishing sensitivity to antibiotics [8]. In the instance of any extrapulmonary TB, a sputum examination and a chest radiograph should be performed as there is a high risk of pulmonary infection [1]. A normal chest film does not reduce clinical suspicion of spinal TB.

Medical therapy is the mainstay of treatment and anti-tuberculous therapy should begin as early as possible [8]. Surgical intervention is reserved for patients who do not respond to medical therapy or who have progressive deformity, instability, or neurologic deficits and for whom diagnostic percutaneous biopsy has failed or is not available [1,8]. In patients with complicated spinal TB, as in the presented case, the combination of medical therapy and surgery yield optimum results [2]. The prognosis is usually good, once the diagnosis has been made and treatment initiated. The pain relief and neurological improvement follow anti-tuberculous therapy in most patients, within three months of the onset of treatment [8].

## Conclusions

Spinal TB is a truly challenging diagnosis that can lead to multiple disabling complications. Due to the typical prolonged and nonspecific clinical picture, a high index of suspicion is necessary to deliver its true diagnosis and, mainly, its etiology. On the other hand, it is vital to establish a treatment in time, to prevent significant consequences, like paraplegia and hypoesthesia, due to spine TB.

In this way, the family doctor plays a fundamental role in the initial investigation, to consider possible differential diagnoses and thus, avoid diagnostic and treatment delays. The family doctor also has a crucial role in the follow-up of these patients, namely in the adherence to prolonged treatment and in the management of the complications of this disease that affect the quality of daily life.
